# Parnassin, a Novel Therapeutic Peptide, Alleviates Skin Lesions in a DNCB-Induced Atopic Dermatitis Mouse Model

**DOI:** 10.3390/biomedicines11051389

**Published:** 2023-05-08

**Authors:** Jeon Hwang-Bo, Karpagam Veerappan, Hyunhye Moon, Tae-Hoon Lee, Kang-Woon Lee, Junhyung Park, Hoyong Chung

**Affiliations:** 1 3BIGS Co., Ltd., Hwaseong 18469, Republic of Korea; jhwangbo@3bigs.com (J.H.-B.); karpagam@3bigs.com (K.V.); hhmoon@3bigs.com (H.M.); jhpark@3bigs.com (J.P.); 2 Department of Applied Chemistry, Kyung Hee University, Yongin 17410, Republic of Korea; thlee@khu.ac.kr; 3 Holoce Ecosystem Conservation Research Institute, Hweongsung 25257, Republic of Korea; holoce@hecri.re.kr

**Keywords:** *Parnassius bremeri*, atopic dermatitis, parnassin, peptide drug, transcriptome

## Abstract

Atopic dermatitis (AD) is a chronic inflammatory skin disease which requires continuous treatment due to its relapsing nature. The current treatment includes steroids and nonsteroidal agents targeting inflammation but long-term administration causes various side effects such as skin atrophy, hirsutism, hypertension and diarrhea. Thus, there is an unmet need for safer and effective therapeutic agents in the treatment of AD. Peptides are small biomolecule drugs which are highly potent and remarkably have less side effects. Parnassin is a tetrapeptide with predicted anti-microbial activity curated from *Parnassius bremeri* transcriptome data. In this study, we confirmed the effect of parnassin on AD using a DNCB-induced AD mouse model and TNF-α/IFN-γ-stimulated HaCaT cells. In the AD mouse model, topical administration of parnassin improved skin lesions and symptoms in AD mice, such as epidermal thickening and mast cell infiltration, similar to the existing treatment, dexamethasone, and did not affect body weight, or the size and weight of spleen. In TNF-α/IFN-γ-stimulated HaCaT cells, parnassin inhibited the expression of Th2-type chemokine CCL17 and CCL22 genes by suppressing JAK2 and p38 MAPK signaling kinases and their downstream transcription factor STAT1. Parnassin also significantly reduced the gene expression of TSLP and IL-31, which are pruritus-inducing cytokines. These findings suggested that parnassin alleviates AD-like lesions via its immunomodulatory effects and can be used as a candidate drug for the prevention and treatment of AD because it is safer than existing treatments.

## 1. Introduction

Atopic dermatitis (AD) is a pruritic eczematous relapsing skin disease affecting 20% of the pediatric and 10% of the adult population globally. The onset of AD usually occurs at infancy and complete remission occurs during childhood; in some cases, it may relapse until adulthood. In adults, both persistent and new onset forms are common, illustrating its chronic nature [1,2,3,4]. The etiology of AD is complex and multifactorial including genetic and environmental factors. Impaired skin barrier function and dysregulated skin inflammation are the profound attributes of AD pathogenicity. Itch is a common symptom of AD which provokes the scratching of the skin causing defective epidermal barrier which leads to the entry of allergens/irritants; eventually the allergen stimulates the release of chemokines (CCL17/TARC and CCL22/MDC) and alarmins (IL-25, IL-33 and TSLP). Consecutively, chemokine and alarmins acts as chemoattractants for T cells; specifically, it activates Th2-driven inflammation by recruiting resident innate group 2 lymphoid cells (ILC2) along with the production of IL-4, IL-5, IL-13 and IL-31. IL-4, IL-13 and IL-31 are pruritogens and their release in turn amplifies the scratching behavior and exacerbates skin barrier disruption. This vicious cycle between enhanced type 2 inflammation and epithelial barrier dysfunction is responsible for pathophysiology of AD [5,6,7,8,9,10].

Management of AD requires a targeted therapy to control adverse immune response and repair skin barrier defect. Currently, both topical (emollients, topical corticosteroids, antibiotics) and systemic (corticosteroids, dupilumab and other immunosuppressants) therapy are in use as mainstay treatments [3,11,12,13]. However, AD is often relapsing in moderate and severe AD, where topical treatment become inadequate and continuous systemic administration of steroids and immunosuppressants is required to decrease the disease burden. However, prolonged use of systemic steroids and immunosuppressants has adverse effects [14,15]. Consequently, the unmet need for effective and safe therapy particularly in treating the pediatric population and moderate/severe form of AD still exists. Peptide drugs are attracting interest in recent years in many therapeutic avenues because of their efficacy, low synthesis cost, and the fact that they are less immunogenic than high-molecular-weight biologicals. AES16-2M is a bioactive short peptide consisting of only five amino acids which showed wound healing effects and attenuates AD-like symptoms [16,17]. Anti-microbial peptide or host defense peptide human β-defensins (hBD-3) has promoted skin barrier function in AD skin in vitro in addition to immunomodulatory activities [18]. Hakuta et al. showed the anti-inflammatory effect of collagen tripeptide in AD patients [19].

Parnassin (KTFR) is a tetrapeptide with anti-bacterial activity curated from *Parnassius bremeri* (*P. bremeri*, red-spotted Apollo butterfly) transcriptome data using our in silico therapeutic peptide screening platform [20]. Therapeutic peptides with the anti-microbial property have also been shown to possess immunomodulatory effects and wound healing efficacy [21,22]. In this study, we evaluated the effect of parnassin on AD and confirmed its mechanism of action using a DNCB-induced AD mouse model and TNF-α/IFN-γ-stimulated HaCaT cells.

## 2. Materials and Methods

### 2.1. Peptide Discovery

Parnassin is a novel therapeutic peptide deciphered from *P. bremeri* transcriptome data using our in silico curation pipeline [20]. Briefly, total RNA was isolated from larva, sequenced and de novo assembled. Peptides were trans decoded from the assembled transcript. The decoded peptides were then subjected to a therapeutic peptide filtration pipeline which includes screening for the physicochemical properties (length, pI, charge), aggregation propensity (in vivo, in vitro), anti-microbial region prediction. The cut-off values used for the physicochemical property prediction are stated in Table 1. Novelty of the peptide was tested using BLAST against three AMP databases: CAMP (Collection of Anti-Microbial Peptides) [23], ADAM (A Database of Anti-Microbial Peptides) [24] and APD (The Anti-microbial Peptide Database) [25], and anti-inflammatory database [26].

### 2.2. Peptide Synthesis

Parnassin was synthesized and purified at Lugen Sci Co., Ltd. (Bucheon, Republic of Korea) according to the methods described previously [20]. Parnassin was dissolved in distilled phosphate buffered saline (PBS) at a concentration of 1 mg/mL.

### 2.3. Animal Study

This study (IACUC2204-005, 13 October 2022) was reviewed and approved by the Institutional Animal Care and Use Committee of Woojung Bio (Hwaseong, Republic of Korea) and animal care and experimental procedures followed the guidelines of Woojung Bio for the care and use of laboratory animals. Six-week-old male SKH-1 mice (BALB/c hairless mice) were purchased from Orient Bio Inc. (Seongnam, Republic of Korea). All mice were maintained at the animal facility of Woojung Bio and housed in an environmentally controlled room with a 12 h light/dark cycle and allowed free access to water. 2,4-Dinitrochlorobenzene (DNCB; Sigma-Aldrich, St. Louis, MO, USA) was dissolved in vehicle (3:1, acetone: olive oil) and used as a sensitizer for inducing AD-like skin lesions in mice. Mice were divided into 6 groups with 5 mice per group: vehicle (control), DNCB, DNCB plus topical treatment of 0.5 mg/kg and 5 mg/kg of parnassin and DNCB plus topical treatment of 0.5 mg/kg and 5 mg/kg of dexamethasone. Mice were treated with 100 µL of 2% of DNCB at a constant time every day for 3 days. After a week, the induction of contact dermatitis was confirmed, DNCB was lowered to 0.5% and 100 μL was treated every 2 days along with parnassin and dexamethasone for 2 weeks. The skin condition was observed weekly and imaged using a digital camera during the experiment. After treatment completion, all mice were euthanized using CO_2_ gas inhalation. Spleens and lymph nodes from each group of mice were collected and weighed. Dorsal skin lesions were collected and fixed with 10% neutral buffered formalin for histological analysis.

### 2.4. Histological Analysis

Fixed skin lesions were embedded into paraffin and sectioned to a thickness of 5 μm. Paraffin sections were stained with hematoxylin/eosin solution or toluidine blue O solution for detecting epidermal thickness or infiltration of mast cells. All sections were digitized under 400× objective magnification and images were captured. Epidermal thickness was measured using the ImageJ program (NIH, version 1.51j8). Mast cells in toluidine blue-stained sections were counted in three different parts.

### 2.5. Cell Culture

HaCaT (Immortal human keratinocyte) cell line was purchased from Korean Cell Line Bank (Seoul, Republic of Korea) and cultured in Dulbecco’s modified Eagle’s medium (DMEM; WELGENE Inc., Daegu, Republic of Korea) supplemented with 10% heat-inactivated fetal bovine serum (FBS; Corning, NY, USA) and 1% penicillin–streptomycin (Gibco, New York, NY, USA) in a humidified incubator at 37 °C and 5% CO_2_.

### 2.6. Cell Viability Assay

HaCaT cells were seeded and incubated at 5 × 10^3^ cells/well in a 96-well plate for 24 h. Cells were treated with different concentrations (0, 1.25, 2.5, 5, 10, 20 μg/mL) of parnassin for 24 h. Cell viability was measured by WST assay using EZ-Cytox (Dogen, Seoul, Republic of Korea) according to the manufacturer’s instructions. A total of 10 μL of EZ-Cytox was added to each well and the plate was incubated at 37 °C for 3 h. The optical density (OD) was measured at 450 nm wavelength using an INNO microplate Spectrophotometer (LTek, Seongnam, Republic of Korea). Cell viability was represented as the percentage of live cells in the parnassin-treated group versus a control group.

### 2.7. Real-Time Quantitative PCR (RT-qPCR) Analysis

HaCaT (3 × 10^5^ cells/well) were cultured in 6-well plates and treated with 10 ng/mL of recombinant human TNF-α (R&D systems, Minneapolis, MN, USA) and 10 ng/mL of recombinant human IFN-γ (R&D systems, MN, USA) in the presence or absence of parnassin (5, 10 μg/mL) and dexamethasone (5, 10 μg/mL) for 24 h. Total RNA isolation was performed using Bio-Zol reagent (Biosesang, Seongnam, Republic of Korea) following manufacturer’s instructions. A total of 1 μg of total RNA was used for cDNA synthesis with oligo dT primer and RevertAid reverse transcriptase (Thermo Fisher Scientific, Waltham, MA, USA). The cDNA was used for amplification of CCL17, CCL22, TSLP, and IL-31 mRNAs with gene specific primer (Table 2) using EzAmp FAST qPCR 2X Master Mix (ELPIS Biotech, Daejeon, Republic of Korea) following manufacturer’s protocol. GAPDH mRNA was used as an internal control. The CCL17, CCL22, TSLP and IL-31 mRNA levels were normalized by GAPDH levels, then relative mRNA expression was calculated by 2^−ΔΔCt^ method.

### 2.8. Western Blot Analysis

HaCaT cells were pretreated with parnassin for 1 h and exposed to TNF-α/IFN-γ (10 ng/mL) for 30 min. Cells were harvested and lysed with 200 µL of RIPA buffer (Biosesang, Seongnam, Republic of Korea). Total protein concentrations were quantified using a Bradford assay reagent (Biosesang, Seongnam, Republic of Korea). Equal amounts of total protein (40 µg) were separated using 10% SDS-PAGE and transferred onto nitrocellulose membrane (Bio-Rad Laboratories, Hercules, CA, USA). Membranes were incubated in blocking solution (5% skim milk in TBS containing 0.05% tween 20) for 1 h and incubated overnight at 4 °C with primary antibodies (anti-JAK2, anti-p-JAK2, anti-p38, anti-p-p38, anti-STAT1, anti-p-STAT1, anti-GAPDH) (Cell signaling, Danvers, MA, USA) diluted 1:1000 in blocking solution. Then, the membranes were washed with TBS-T, and probed with peroxidase-conjugated anti-mouse-IgG and anti-rabbit-IgG antibodies (cell signaling) at a 1:5000 dilution in a blocking. Protein bands were detected using ECL chemiluminescence reagents (Amersharm Phamacia Biotech, Piscataway, NJ, USA).

### 2.9. Statistical Analysis

All data were represented as mean ± SD. Student’s t-test and one-way ANOVA were used to evaluate the significance between groups (^#^ *p* < 0.05, ^##^ *p* < 0.01, ^###^ *p* < 0.001 vs. control group; * *p* < 0.05, ** *p* < 0.01, *** *p* < 0.001 vs. DNCB group).

## 3. Results

### 3.1. Effect of Parnassin in a DNCB-Induced AD Mouse Model

**Figure 1:**
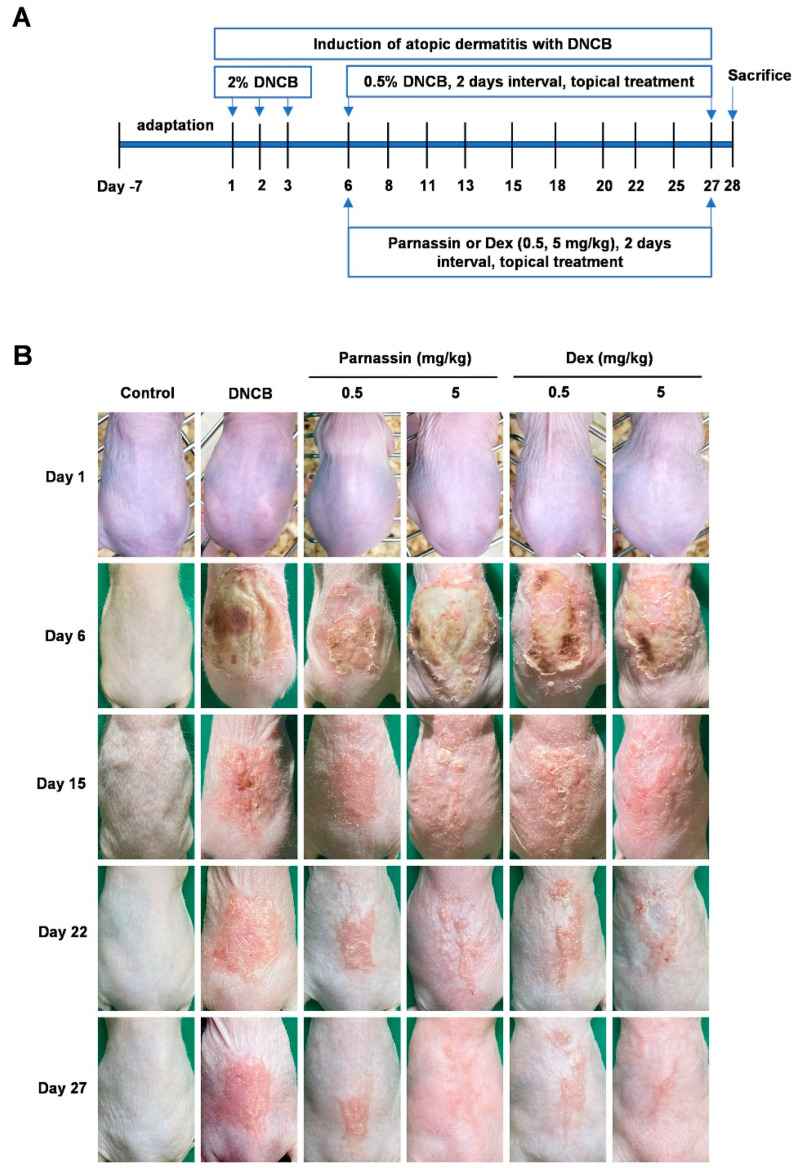
Establishment of a DNCB-induced AD mouse model and the effects of parnassin in the mouse model. (**A**) Schematic diagram of the experimental design. AD-like skin lesions were induced by treating mice with 2% DNCB for 3 days. One week after induction, topical application of 0.5% DNCB was treated every 2 days along with parnassin and dexamethasone to the lesion site. All mice were euthanized at day 28. (**B**) Clinical severity of inflammatory skin lesions. Photographs were taken on day 1, 6, 15, 22, and 27.

To investigate the in vivo effect of parnassin on AD, a DNCB-induced AD-like contact dermatitis mouse model was used. As shown in Figure 1A, DNCB treatment (day 1, 2, 3), sensitized the dorsal skin to AD-like lesions and it was confirmed that skin lesions were induced by DNCB in all groups except for the control group at day 6. Then, parnassin and dexamethasone were treated on alternate days at the doses of 0.5 and 5 mg/kg. In the peptide treatment group, skin inflammation symptoms were significantly alleviated from a short treatment period of about one week, and skin inflammation symptoms were alleviated and the skin surface was observed similar to that of the control group at the end of 3 weeks of treatment (Figure 1B).

**Figure 2:**
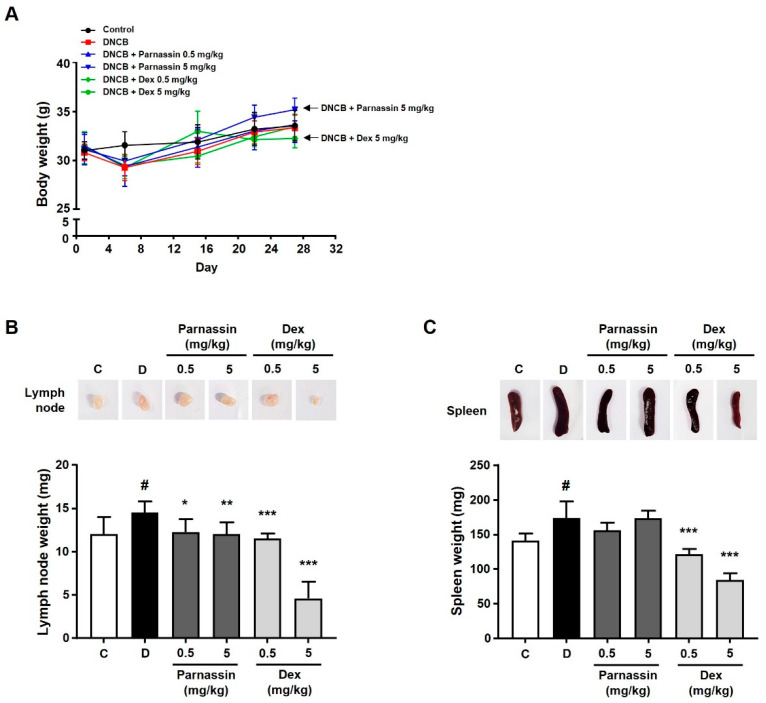
Effect of parnassin on body weight and organ weight of DNCB-induced AD mouse model. (**A**) Body weights of all mice were measured on day 1, 6, 15, 22, and 27. (**B**,**C**) Size comparison of draining lymph node (**B**) and spleen (**C**) by photographic images. Spleens and lymph nodes were isolated from all groups on day 28. Spleens and lymph nodes from each group of mice were collected and weighed. The spleen and lymph node weights of each group were represented as a bar diagram. Data are presented as a mean ± SD (^#^ *p* < 0.05 vs. control group; * *p* < 0.05, ** *p* < 0.01, *** *p* < 0.001 vs. DNCB group).

The body weight change was examined among the control group, the DNCB-treated group, the parnassin-treated group, and the dexamethasone-treated group: no noticeable body weight change was observed in the other groups except for the 5 mg/kg dexamethasone-treated group. However, body weight loss was observed in the 5 mg/kg dexamethasone-treated group (Figure 2A).

To confirm the effect of the parnassin on the spleen and lymph nodes, which are organs mainly involved in the immune response, the spleen and lymph nodes were collected and compared in size and weight. In the DNCB-treated group, immune response was stimulated, in consequence, the size and weight of the spleen and lymph nodes were significantly increased compared with the control group. In the parnassin-treated group, there was no significant effect on the size and weight of the spleen compared to the DNCB-treated group, but the size and weight of the lymph nodes were significantly reduced. In the 0.5 mg/kg dexamethasone-treated group, the size and weight of the lymph node and spleen decreased to a level similar to that the control group. In the 5 mg/kg dexamethasone-treated group, both the size and weight of the spleen and lymph node were abnormally reduced (Figure 2B,C).

**Figure 3:**
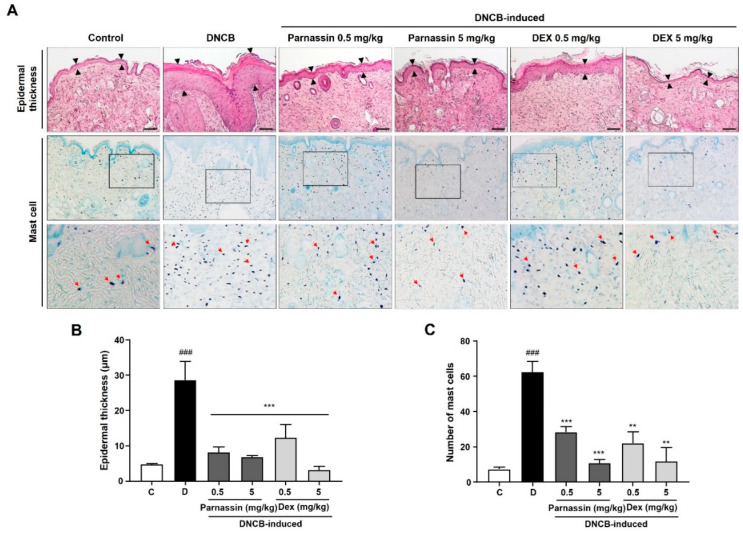
Effect of parnassin on the epidermal thickness of the dorsal skin and mast cell infiltration. (**A**) Mouse back skin lesions were fixed with 10% neutral buffered formalin, sectioned to a thickness of 5 μm and stained with hematoxylin and eosin and toluidine blue O stain. All stained sections were digitalized, and images were captured under 400× objective magnification. Scale bar = 100 μm. Arrow indicates mast cells (**B**) Epidermal thickness was measured using the Image J program. (**C**) Number of mast cells were counted. Data were represented as a bar diagram. (^###^ *p* < 0.001 vs. control group; ** *p* < 0.01 and *** *p* < 0.001 vs. DNCB group).

One of the most observed histological features of AD includes the thickening of the epidermis due to hyperkeratosis [27]. To further evaluate the effect of parnassin on histological changes of lesioned skin, histopathological changes were investigated using H&E and toluidine blue O staining. Skin sections of DNCB-treated mice showed abnormal thickening of the epidermis due to epidermal hyperplasia and severe keratinization (including hyperkeratosis) compared to mice in the control group. Parnassin administration markedly improved DNCB-induced histological changes. As a result, the histological appearance of the skin was similar to that of the control group (Figure 3A,B). The histological changes induced by DNCB were also greatly recovered in dexamethasone-treated group, but it was confirmed that the epidermal thickness in the 5 mg/kg dexamethasone-treated group was abnormally thinner than the control group.

Mast cell infiltration was examined with toluidine blue O staining, and it was significantly increased in the skin lesions of DNCB-treated mice. Topical administration of parnassin and dexamethasone inhibited DNCB-induced mast cell infiltration in the lesional skin area. Parnassin inhibited mast cell infiltration similarly to dexamethasone (Figure 3A,C).

### 3.2. Effect of Parnassin on CCL17 and CCL22 mRNA Expression in TNF-α/IFN-γ-Stimulated HaCaT Cells

**Figure 4:**
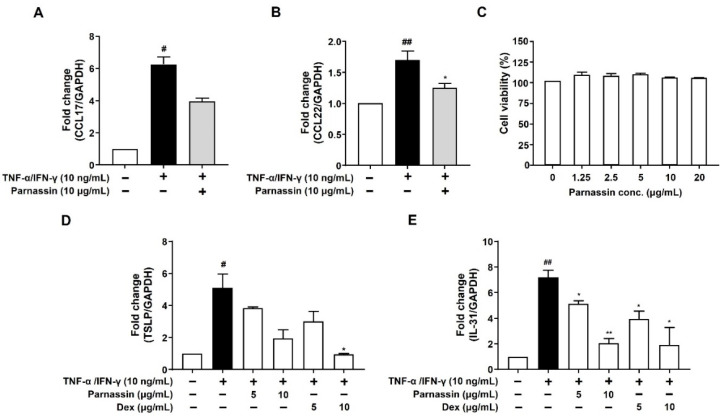
Effect of parnassin on mRNA expression of CCL17, CCL22, TSLP, IL-31 in TNF-α/IFN-γ-stimulated HaCaT cells. (**A**,**B**) HaCaT cells were pretreated with 10 μg/mL of peptide for 1 h and stimulated with TNF-α/IFN-γ for 24 h. Total RNA was isolated, and CCL17 and CCL22 mRNA were analyzed by RT-PCR using specific primers. GAPDH was used as the internal control. (**C**) HaCaT cells were treated with peptide at the indicated concentrations for 24 h. Cell viability was measured by WST assay. (**D**,**E**) Total RNA was isolated, and TSLP and IL-31 mRNA levels were analyzed by RT-qPCR using specific primers. The mRNA levels were normalized by GAPDH levels. Data are presented as a mean ± SD of three independent experiments. (^#^ *p* < 0.05, ^##^ *p* < 0.01 vs. control; * *p* < 0.05, ** *p* < 0.01 vs. TNF-α/IFN-γ-stimulated HaCaT cells).

AD is a Th2-dominant inflammatory skin disease and an abnormal expression of Th2 chemokines such as TARC/CCL17 (thymus and activation-regulated chemokine) and MDC/CCL22 (macrophage-derived chemokine) in keratinocytes were observed at the inflammatory site of epidermis [28]. Therefore, we investigated the effects of parnassin on the expression of CCL17 and CCL22 in TNF-α/IFN-γ-stimulated HaCaT cells. In the TNF-α/IFN-γ treatment groups, the mRNA expression levels of CCL17 and CCL22 were significantly increased compared with the untreated control group, and the increased mRNA levels of CCL17 and CCL22 were decreased by the parnassin treatment (Figure 4A,B). These results show that the parnassin can ameliorate Th2-dominant AD by inhibiting the mRNA expression of CCL17 and CCL22 induced by TNF-α/IFN-γ in HaCaT cells.

To confirm that the inhibitory effect of the parnassin on CCL17 and CCL22 expression was not due to the cytotoxicity of the peptide, we investigated the effect of the parnassin on the viability of HaCaT cells using the WST assay. The results confirmed that the peptide did not affect the viability of HaCaT cells at all tested concentrations (Figure 4C).

### 3.3. Effect of Parnassin on TSLP and IL-31 mRNA Expression in TNF-α/IFN-γ-Stimulated HaCaT Cells

TSLP and IL-31 are pruritogenic cytokines secreted from keratinocytes. The expression of TSLP and IL-31 is elevated in the lesional skin of AD patients but not in other dermatitis. Thus, TSLP and IL-31 are considered specific cytokine markers for AD [29,30]. To confirm the effect of the parnassin on these cytokines, we investigated the expression of TSLP and IL-31 in TNF-α/IFN-γ-stimulated HaCaT cells, using RT-qPCR analysis. In the group treated with TNF-α/IFN-γ, the mRNA expression levels of TSLP and IL-31 were increased by 411% and 619%, respectively, compared to the untreated control group. Increased TSLP and IL-31 mRNA levels were considerably reduced by 62% and 82.9% upon 10 μg/mL parnassin treatment, respectively. In addition, the pruritogen inhibitory effect of the parnassin was similar to that of dexamethasone at the same concentration which is used as a positive control (Figure 4D,E).

### 3.4. Effect of Parnassin on the Activation of JAK2, p38 MAPK, and STAT1 in TNF-α/IFN-γ-Stimulated HaCaT Cells

To elucidate the mechanism of inhibition of Th2 chemokines CCL17 and CCL22 production by parnassin in keratinocytes, we investigated the signaling molecules implicated in their production. TNF-α and IFN-γ activate transcription factor STAT1 to induce the production of TARC/CCL17 and MDC/CCL22 [31]. Evidently, STAT1 binding sequences were found in the TARC and MDC promoter regions [32]. In addition, these transcription factors are activated by upstream signaling molecules such as MAP kinase and JAK2 [28,33,34].

**Figure 5:**
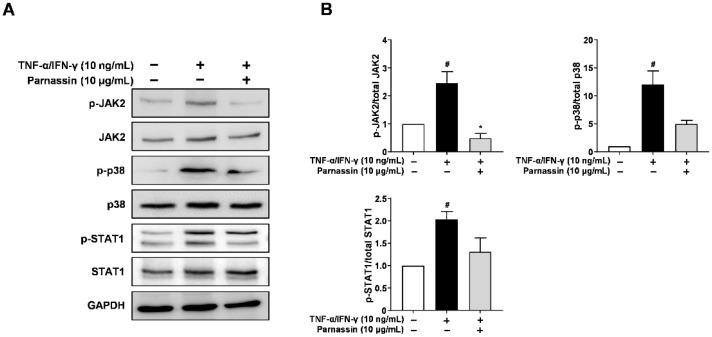
Effect of parnassin on activation of JAK2, p38 and STAT1. (**A**) Cells were pretreated with peptide 1 h and stimulated TNF-α/IFN-γ for 30 min. Total proteins were subjected to Western blot using specific antibodies for JAK2, p38, STAT1 and their respective phosphorylated forms. (**B**) Relative intensity graphs of total and phosphorylated JAK2, p38, STAT1. (^#^ *p* < 0.05 vs. control; * *p* < 0.05 vs. TNF-α/IFN-γ-stimulated HaCaT cells).

Firstly, we investigated the phosphorylation levels of JAK2 and p38 MAPK using Western blot. As shown in Figure 5, in TNF-α/IFN-γ-stimulated HaCaT cells, phosphorylation of JAK2 and p38 MAPK was increased. Parnassin treated group inhibited the increased phosphorylation of JAK2 and p38 MAPK. These results show that JAK2 and p38 MAPK are involved in CCL17 and CCL22 expression in HaCaT cells stimulated by TNF-α/IFN-γ, and the phosphorylation of p38 MAPK and JAK2 is inhibited by parnassin treatment.

We next investigated the effect of peptide on the STAT1, downstream transcription factors, in TNF-α/IFN-γ-stimulated HaCaT cells. STAT1 is phosphorylated by JAK2 in response to IFN-γ and translocated to the nucleus [28]. Phosphorylation of STAT1 increased in TNF-α/IFN-γ-stimulated HaCaT cells. The increased phosphorylation of STAT1 was decreased by parnassin treatment. These results show that parnassin inhibited the expression of Th2-type chemokine CCL17 and CCL22 genes by suppressing JAK2 and p38 MAPK signaling kinases and their downstream transcription factor STAT1 (Figure 5).

## 4. Discussion

Atopic dermatitis is a chronic inflammatory relapsing skin disease that requires continuous management and treatment. Topical corticosteroids have been used as effective AD therapy for over 50 years. Anti-inflammatory and immunosuppressive effects of the topical steroids are their major mechanisms of action in treating inflammation-associated skin disease such as AD. However, their long-term use causes various side effects such as skin atrophy, depigmentation, perioral dermatitis, tachyphylaxis and if it is used infants can cause symptoms of growth retardation, osteoporosis [35]. Recently, the use of nonsteroidal agents such as cyclosporin A and tacrolimus is increasing. However, non-steroidal drugs also have various side effects such as renal impairment, hirsutism, hypertension and diarrhea and therefore continuing treatment is difficult. Thus, there is an unmet need for a new therapeutic agent that has an indispensable effect in treating AD without adverse side effects.

Peptide drugs are highly selective to the target, and at the same time, they are relatively safe because they have negligible binding affinity to substances other than the target and are less toxic because they hardly accumulate in living tissues [36]. Taking advantage of these properties of peptide drugs, we have selected parnassin, a novel peptide drug deciphered from red spotted apollo butterfly and evaluated the in vivo and in vitro efficacy.

Under normal skin conditions, the keratinocytes have the same rate of regeneration and loss, so that a constant thickness of the stratum corneum is maintained. However, in abnormal skin conditions such as AD, the stratum corneum is thickened by the exfoliation of keratinocytes, and dry skin is induced by tissue collapse due to water loss [37]. Additionally, AD is commonly characterized by dry, itchy eczematous skin lesions along with the infiltration of immune cells [28].

To confirm the in vivo effect of parnassin, we used a DNCB-induced AD mouse model, which can induce dermatitis within a relatively short period and is easy to visually check skin symptoms such as lichen and blisters. In this study, topical application of DNCB on the back of mice induced many of the clinical symptoms of AD and the skin lesions of mice were similar to those of patients with AD. Epidermal thickening was clearly observed in the skin lesions of DNCB-treated mice, and the DNCB-induced epidermal thickening was greatly reduced both in the dexamethasone and parnassin-treated groups. However, in the 5 mg/kg dexamethasone-treated group, skin atrophy, a typical side effect of topical corticosteroid administration, was observed. Concurrently, parnassin administered at the same concentration significantly reduced DNCB-induced epidermal thickening but did not exhibit skin atrophy. Parnassin inhibited mast cell infiltration similarly to dexamethasone.

In addition, topical as well systemic administration of dexamethasone reduces the spleen and body weight of laboratory animals, and such a decrease in spleen and body weight is generally recognized as one of the side effects caused by the suppression of immune function [38]. In this study, it was confirmed that parnassin exhibited similar efficacy to dexamethasone for AD in experimental animals. However, 5 mg/mL dexamethasone treatment reduced body weight and spleen size and weight, which were significantly lower than those of the control group. In contrast, parnassin treatment at the same concentration did not affect body weight or spleen size and weight. These results indicate that parnassin has a similar level of AD treatment efficacy without the side effects of existing treatments.

Many previous studies have shown that IFN-γ and TNF-α stimulate epidermal keratinocytes to activate many signaling pathways and participate in the promotion of inflammation [39,40,41]. Therefore, in this study, we used HaCaT cells stimulated with TNF-α and IFN-γ to determine the in vitro effect of the parnassin on AD. Many studies have shown that chemokines TARC/CCL17 and MDC/CCL22 have been increased in serum and/or lesional tissues of AD patients and animal models of AD [10,42,43,44,45]. TARC and MDC bind to CC chemokine receptor 4 (CCR4), and this interaction plays an important role in migrating Th2 cells to inflamed tissues. It has been reported that the serum concentrations of MDC and TARC are closely correlated with Th2-inflammation-driven skin diseases such as AD [45]. Serum concentrations of MDC and TARC were high in patients with AD, and treatment with cyclosporine A or topical corticosteroids significantly reduced TARC/CCL17 and MDC/CCL22 concentrations and ameliorated the disease [9]. Collectively, CCL17 and CCL22 can be regarded as representative chemokine targets for AD drug efficacy evaluation. Therefore, down-regulating TARC and MDC production in keratinocytes could be an important anti-inflammatory strategy. Thus, we investigated the effect of parnassin on the expression of TARC and MDC in TNF-α/IFN-γ-stimulated HaCaT cells. Parnassin significantly decreased the expression of MDC and TARC induced by TNF-α and IFN-γ stimulation in HaCaT cells.

TSLP and IL-31 are Th2 cytokines that induce pruritus by activating skin somatosensory neurons directly or indirectly through stimulation of immune cells and are highly up-regulated in patients with AD [46]. These are representative atopic cytokines that promote pathological progression through the breakdown of the skin barrier and exacerbation of itch leading to amplification of inflammation [47]. We investigated the effect of parnassin on the expression of TSLP and IL-31 in TNF-α/IFN-γ-stimulated HaCaT cells. Similar to chemokines, parnassin inhibited TSLP and IL-31at the transcription level in TNF-α- and INF-γ-stimulated HaCaT cells. The inhibition of the expression of TSLP and IL-31 in parnassin-treated HaCaT cells was similar to that of dexamethasone used as a positive control.

**Figure 6:**
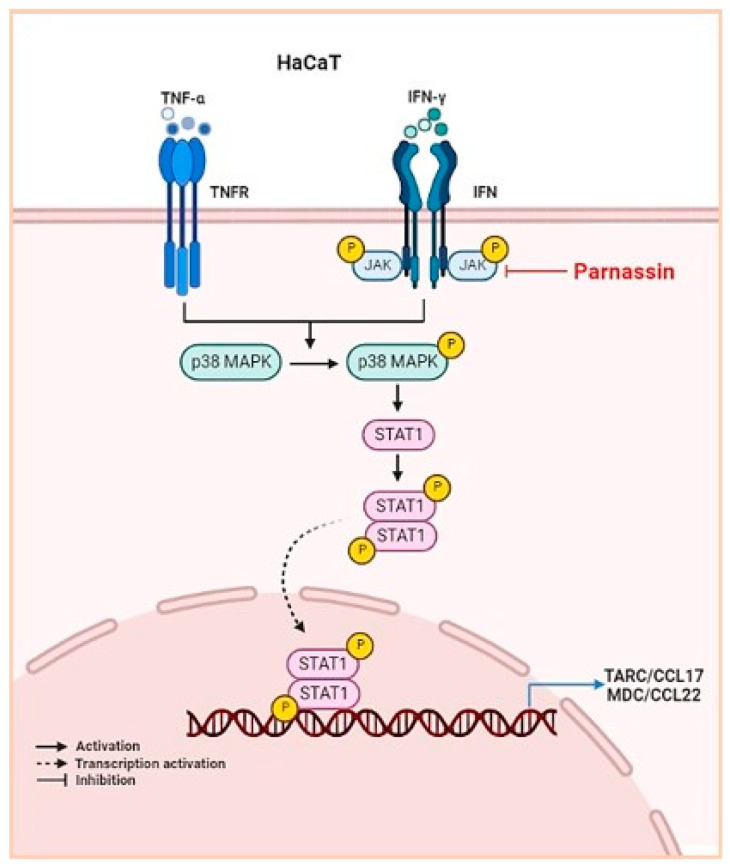
Schematic illustration of the mechanism of action of parnassin.

In HaCaT cells, TNF-α and IFN-γ activate JAK2, p38 MAPK and Raf-a by phosphorylation after binding to their dedicated receptors [48,49]. Subsequently, activated JAK2 and p38 MAPK phosphorylate transcription regulator STAT1. Unphosphorylated STATs are present in the cytoplasm and upon activation translocate into the nucleus where they bind to the promoter of CCL17 and CCL22 to trigger its synthesis [31,32,34,50,51]. This indicates that inhibition of STAT1 activity down regulates the production of CCL17 and CCL22. In this study, we confirmed that JAK2, p38 MAPK and STAT1 were rapidly phosphorylated by TNF-α/IFN-γ stimulation in HaCaT keratinocytes, and that the phosphorylation of JAK2, p38 MAPK and STAT1 was inhibited by parnassin treatment. These results showed that parnassin exhibits its anti-inflammatory activity and attenuates AD-like symptoms by inhibiting JAK2 and p38 MAPK signaling kinases and their downstream transcription factors STAT1 (Figure 6).

Currently, JAK inhibitors are used for various inflammatory diseases, but safety issues have been reported. In the case of parnassin, it is considered to be much safer than other JAK inhibitors currently in use because it has the advantage of being a peptide drug with high safety and selectivity. In addition, unlike other JAK inhibitors, it is thought to be much more effective by inhibiting not only JAK2 but also p38.

Taken together, in a DNCB-induced AD mouse model, parnassin was found to show a similar level of AD treatment efficacy as the existing treatment, dexamethasone, without side effects. In TNF-α/IFN-γ-stimulated HaCaT cells, parnassin inhibited JAK2 and p38 MAPK signaling kinases through inhibition of STAT1, thereby suppressing the expression of the Th2-type chemokine CCL17 and CCL22 genes. Parnassin also significantly reduced the gene expression of TSLP and IL-31, which are pruritus-inducing cytokines. These findings suggest that parnassin could be utilized as a therapeutic peptide for the prevention and treatment of AD that is safer and more effective than existing therapies.

## Figures and Tables

**Table 1:** Physicochemical properties of parnassin.

Propensity	Tools	Descriptions/Parameters	Cutoff	Parnassin
Physicochemical	Pepstats	Peptide Length	≥2 to 50	4
	Pepstats	Charge	>0 (+)	2
	Pepstats	Isoelectric Point(pI)	≥8 to ≤12	11.65
	AMPA	Stretch	≥1	1
Aggregation (In vitro)	Aggrescan	Na4vSS	≥−40 Na4vSS ≤60	−38.6
Aggregation (In vivo)	Tango	AGG	≤500	0
	Tango	Helix	≥0 Helix ≤25	0
	Tango	Beta	≥25 Beta ≤100	30.81

+ denotes positive charge.

**Table 2:** List of RT-qPCR primer pairs.

Genes	Specific Primer Sequences	
	Forward (5′-3′)	Reverse (5′-3′)
CCL17	ACTGCTCCAGGGATGCCATCGTTTTT	ACAAGGGGATGGATCTCCCTCACTG
CCL22	AGGACAGAGCATGGCTCGCCTACAGA	TAATGGCAGGGAGGTAGGGCTCCTGA
TSLP	GGGGCTAAACCATGACAGAA	GTTTGGCTGAAGGCTTGTTC
IL-31	CGACGTCTGTGCTCTTTCTG	AGCATCTTCGAGAGGGACTG
GAPDH	GACCCTCGAAATCCCATCACAG	GTGCGAACTTCCACGGTGTGTT

## Data Availability

All data generated or analyzed during this study are included in this published article.
